# Comparative studies of three cholesteryl ester transfer proteins and their interactions with known inhibitors

**DOI:** 10.1371/journal.pone.0180772

**Published:** 2017-08-02

**Authors:** Ziyun Wang, Manabu Niimi, Qianzhi Ding, Zhenming Liu, Ling Wang, Jifeng Zhang, Jun Xu, Jianglin Fan

**Affiliations:** 1 Department of Molecular Pathology, Interdisciplinary Graduate School of Medicine, University of Yamanashi, Yamanashi, Japan; 2 State Key Laboratory of Natural and Biomimetic Drugs, School of Pharmaceutical Sciences, Peking University, Beijing, China; 3 School of Pharmaceutical Sciences & Institute of Human Virology, Sun Yat-Sen University, Guangzhou, China; 4 Pre-Incubator for Innovative Drugs & Medicine, School of Bioscience and Bioengineering, South China University of Technology, Guangzhou, China; 5 Cardiovascular Center, Department of Internal Medicine, University of Michigan, Ann Arbor, Michigan, United States of America; 6 Deparment of Pathology, Xi’an Medical University, Xi’an, China; Medizinische Universitat Innsbruck, AUSTRIA

## Abstract

Cholesteryl ester transfer protein (CETP) is a plasma protein that mediates bidirectional transfers of cholesteryl esters and triglycerides between low-density lipoproteins and high-density lipoproteins (HDL). Because low levels of plasma CETP are associated with increased plasma HDL-cholesterol, therapeutic inhibition of CETP activity is considered an attractive strategy for elevating plasma HDL-cholesterol, thereby hoping to reduce the risk of cardiovascular disease. Interestingly, only a few laboratory animals, such as rabbits, guinea pigs, and hamsters, have plasma CETP activity, whereas mice and rats do not. It is not known whether all CETPs in these laboratory animals are functionally similar to human CETP. In the current study, we compared plasma CETP activity and characterized the plasma lipoprotein profiles of these animals. Furthermore, we studied the three CETP molecular structures, physicochemical characteristics, and binding properties with known CETP inhibitors *in silico*. Our results showed that rabbits exhibited higher CETP activity than guinea pigs and hamsters, while these animals had different lipoprotein profiles. CETP inhibitors can inhibit rabbit and hamster CETP activity in a similar manner to human CETP. Analysis of CETP molecules *in silico* revealed that rabbit and hamster CETP showed many features that are similar to human CETP. These results provide novel insights into understanding CETP functions and molecular properties.

## Introduction

Cholesteryl ester transfer protein (CETP) is a hydrophobic glycoprotein synthesized mainly in the liver and circulates in plasma in association with HDL[1]. CETP transports cholesteryl esters from HDLs to apolipoprotein (apo)-B containing particles, therefore playing an important role in the metabolism of lipoproteins and the reverse cholesterol transport from the peripheral tissues to the liver[1]. Patients genetically deficient in the CETP gene showed low or no CETP activity along with hyper-HDL-cholesterolemia[2]. Furthermore, it has been known that high levels of plasma HDL-C are inversely associated with low risk of coronary heart disease (CHD)[3]; thus, elevation of plasma HDL-C levels through inhibition of CETP was also considered an alternative therapy to treat CHD[4]. This notion was initially supported by the finding that therapeutic inhibition of CETP (such as CETP antisense, vaccine, or inhibitors) in experimental animals led to the elevation of plasma HDL-C and the reduction of atherosclerosis[5–9]. However, in human clinical trials, three CETP inhibitors either failed due to excess death (torcetrapib) or were terminated due to insufficient efficacy (dalcetrapib and evacetrapib)[10–12]. Currently, only anacetrapib is still under testing in a Phase III clinical trial[13]. Because it is still controversial regarding whether CETP inhibition is beneficial for the treatment of CHD[14], there is a need to examine the pathophysiological functions of CETP using experimental animals[15]. Human CETP and its interactions with CETP inhibitors have been extensively investigated[16–18]. Interestingly, in addition to humans and other primates, only a few laboratory animals, such as rabbits, guinea pigs, and hamsters, exhibit detectable plasma CETP activity, whereas rodents (mice and rats) do not have endogenous CETP genes[19]. To study pathophysiological roles of CETP in lipid metabolism and atherosclerosis, it is essential to use appropriate animal models with plasma CETP activity. In fact, it is not known whether CETP-possessing mammals have CETP functions similar to those of human CETP. To examine this question, we performed the current study in an attempt to (1) construct three CETP 3-D molecule structures by homology *in silico* and examine possible pockets of these CETP models; (2) compare their CETP activity along with characterization of the plasma lipoprotein profiles; and (3) examine CETP interactions with known inhibitors. Our results indicate that rabbit and hamster CETP but not guinea pig CETP is similar to human CETP in terms of activity and inhibitor interactions.

## Materials and methods

### Molecular phylogenetic analysis of CETP-possessing animals

Through a search on the GenBank, we constructed an evolutionary tree of 8 animals which have CETP genes, including humans, chimpanzees, crab-eating macaques, tree shrews, rabbits, guinea pigs, hamsters, and chickens. The evolutionary history was inferred using the Maximum Likelihood method based on the JTT matrix-based model. The tree with the highest log likelihood (-3907.1590) is shown. Initial trees for the heuristic search were obtained automatically by applying Neighbor-Join and BioNJ algorithms to a matrix of pairwise distances estimated using a JTT model and selecting the topology with the superior log likelihood value. The tree was drawn to scale, with branch lengths measured in the number of substitutions per site. There were a total of 412 positions in the final dataset. Evolutionary analyses were conducted in MEGA v 7.0 software. Furthermore, we compared the CETPs of three laboratory animals (rabbit, guinea pig, and hamster) with human CETP. All CETP sequences were obtained from the PubMed database (www.ncbi.nlm.nih.gov/entrez). Sequence similarity searching was carried out using BLAST searches as reported previously[20, 21].

### *In silico* analyses of CETPs

CETP molecules were constructed, analyzed, and described in **Figures A-F and Tables A-C in** S1 File. The binding pockets of the CETP models were derived from MDS results and further studied using Cavity in the LigBuilder v.2.0 Program to identify protein-binding sites and characterize druggable ligand-binding pockets. It was used to estimate the best binding affinity of each proposed binding pocket. Functions of geometric shape, hydrogen bonding, and hydrophobic effect for each cavity were calculated and expressed as scores. The binding energy of CETP inhibitors (evacetrapib and anacetrapib) to each CETP was compared **(Methods in** S1 File**)**.

### Plasma CETP activity, plasma lipids, and lipoprotein profiles

Male Japanese white rabbits (16 weeks old, n = 5), male golden Syrian hamsters (7 weeks old, n = 5), and male Hartley guinea pigs (11 weeks old, n = 5) were obtained from Japan SLC (Shizuoka, Japan). All animals were fed a standard laboratory diet *ad libitum*. For the determination of plasma lipids and CETP activity, blood was taken after 16 h fasting either from the auricular artery (rabbits) or the abdominal vena cava after being anesthetized with sodium pentobarbital (guinea pigs and hamsters). All animal experiments were performed with the approval of the Animal Care Committee of the University of Yamanashi. Human plasma was obtained from healthy male volunteers in the laboratory (20–40 years old, n = 5) with written informed consent and used for a comparison. The current study was approved by the Yamanashi University ethics committee (No. 1644) and all volunteers were fully aware of the purpose of the current experiment before blood collection.

Plasma CETP activity was assessed using fluorometric assay kits which measured CETP-mediated transfer of the fluorescence-labeled neutral lipids (BioVision, Milpitas, CA, USA). Plasma total cholesterol (TC), triglycerides (TG), and HDL-C were measured using enzymatic assay kits (Wako Pure Chemical, Osaka, Japan)[22]. Plasma lipoprotein profiles were analyzed using agarose gel electrophoresis and high performance liquid chromatography (HPLC)[23]. Plasma (4 μL) was electrophoresed on 1% agarose gel (Helena Laboratories, Saint, Japan) and stained for neutral lipids with Fat Red 7B staining. In addition, plasma lipoproteins were analyzed by HPLC on gel filtration columns at Skylight Biotech (Akita, Japan) as reported before[23].

### *In vitro* CETP inhibition study

To evaluate the inhibitory efficacy of CETP inhibitors on the plasma CETP activity of the three animals and humans, we performed the inhibitory activity assay using a fluorometric assay kit (Roar Biomedical, New York, NY, USA). Briefly, fluorescence-labeled donor particles and serially diluted CETP inhibitors dissolved in DMSO solution were incubated in the presence of each plasma and acceptor particles for 3 h at 37°C. Subsequently, the amount of fluorescent neutral lipids transferred to the acceptor particles was quantified by a fluorescence spectrophotometer Gemini EM (Molecular Devices, Sunnyvale, CA, USA). Torcetrapib was purchased from Sigma-Aldrich (St. Louis, MO, USA), and anacetrapib and evacetrapib were from MedChemExpress (Monmouth Junction, NJ, USA). Dalcetrapib was provided by Roche. Torcetrapib, anacetrapib, and evacetrapib were tested at 0.0312 to 50 nM, and dalcetrapib at 62.5 to 1000 nM, based on the doses reported. The half maximal inhibitory concentration (IC_50_) was calculated using SoftMax Pro software (Molecular Devices, Sunnyvale, CA, USA).

## Results

**Fig 1:**
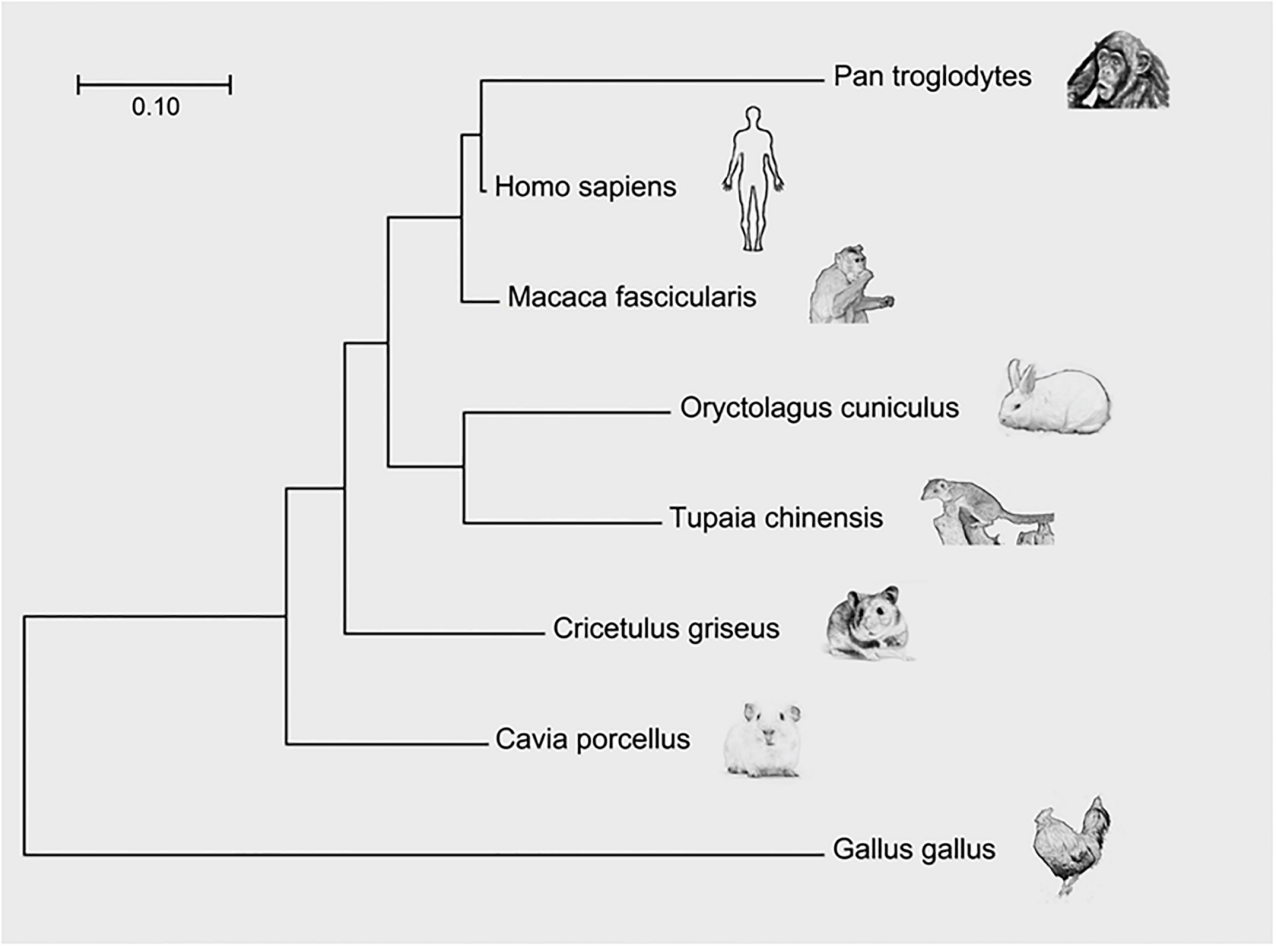
Evolutionary tree of CETP-possessing mammals.

We first constructed an evolutionary tree of eight species that have CETP genes based on a search of GenBank (Fig 1). Among five non-primates, rabbit CETP is the closest to that of primates. We focused on three commonly-used laboratory animals (rabbits, guinea pigs, and hamsters) regarding the CETP gene and protein sequence and compared their similarities with human CETP. CETP proteins in all species are 53 kDa in size, but the rabbit CETP sequence is slightly more identical to human CETP compared with guinea pig and hamster CETP, as summarized in Table 1.

**Table 1:** Comparison of properties of four CETP molecules.

CETP	Chromosome location	DNA (kb)	Exon number	RNA (kb)	cds Identical with Human	Mature protein (kDa)	Identical with Human
Human	16q21 (16:56,961,850–56,983,845)	22	17	1.8 (NM_000078)		53 (NP_000069)	
Rabbit	5:13,183,526–13,200,463	15.9	16	1.99 (XM_002711536)	85%	53 (XP_002711582)	81%
Guinea pig	Unknown	18.1	16	1.5 (XM_003472075)	84%	53 (XP_003472123)	79%
Hamster	Unknown	20.1	17	1.48 (XM_003503614)	84%	53 (XP_003503662)	80%

### CETP molecule structures and their binding pockets

**Fig 2:**
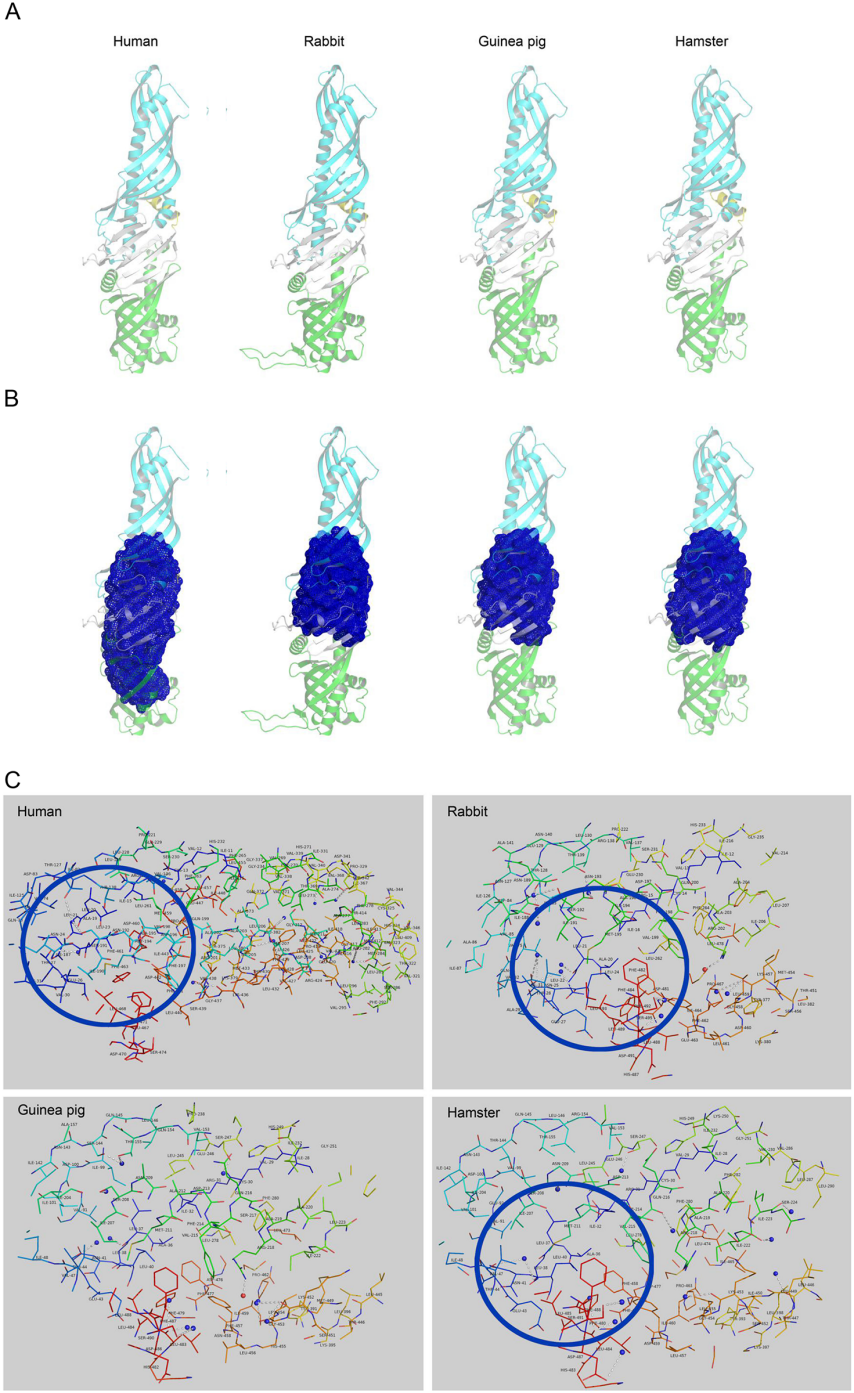
Views of putative binding pockets in human, rabbit, guinea pig, and hamster CETPs. Predicted pockets of the CETP molecules of four species are shown. The binding pocket information was created by the Cavity program. The graphics were generated using the PyMOL program (http://www.pymol.org). **A-B**. 3-D structures of CETP molecules, **C**. the residues of the four CETP pockets. Human, rabbit and hamster CETP residues are highlighted by a round circle.

We next constructed three 3-D CETP molecule models using human CETP[17] as a template (Fig 2). These 3-D models were further stereo-chemically validated using additional parameters such as PROCHECK, and by analyzing residue-by-residue geometry and overall structural geometry **(Figure A in** S1 File**)**. Most of the residues in these proteins were located in allowed regions (> 99%); therefore, these models were acceptable. Using these models, we were specifically interested in elucidating their binding pockets. As described in the Methods, binding pockets were filtered using the following two criteria: high predicted pKd values (~1 nM was used as the cut-off value for judging whether the binding pocket had the potential for achieving high binding affinity) and the location of the central β-sheet. The final proposed binding pocket of each CETP is therefore confirmed for the following structure-based investigations.

We characterized the residues of the four CETP pockets proposed above. In Fig 2, there are twelve hydrogen bond donors (corresponding to Leu23 or Thr27, Thr127 or Ser191, Ile205 or Ser207, Leu206, Ser230, Arg282, Arg282 or Met284, Ser342, Thr369, Val421, Lys436, and Leu467) in the human CETP pocket. In the case of the rabbit, one hydrogen bond acceptor (corresponding to Arg202) and eleven hydrogen bond donors (corresponding to Leu21, Asn25 or Thr28, Leu24 or Thr28, Thr128, Thr128 or Ser192, Thr139 or Ser192, Ile188, Lys457, Lys457, Leu488, and Ser495) were identified. For the guinea pig, there were eleven hydrogen bond donors (corresponding to Leu37, Thr44, Ser144 or Thr151, Ser208, Ser247, Lys452, Lys452, Gly453, Leu483, Leu483, and Ser490), and one hydrogen bond acceptor (corresponding to Arg218) in the binding pocket. For the hamster, there were twelve hydrogen bond donors (corresponding to Leu40 or Thr44, Ser208, Val215, Ile222, Ile223, Ser247, Leu446, Lys453, Leu484, Leu484, Asp487, and Ser491) in the binding pocket. The use of Leu (23 in human, 24 in rabbit, and 40 in hamster) or Thr (27 in human, 28 in rabbit, and 44 in hamster) residues as hydrogen bond donors occurs in 3 different species (Fig 2C). These common features may be crucial for CETP interactions with inhibitors.

### Analysis of plasma CETP activity, lipids, and lipoprotein profiles

**Fig 3:**
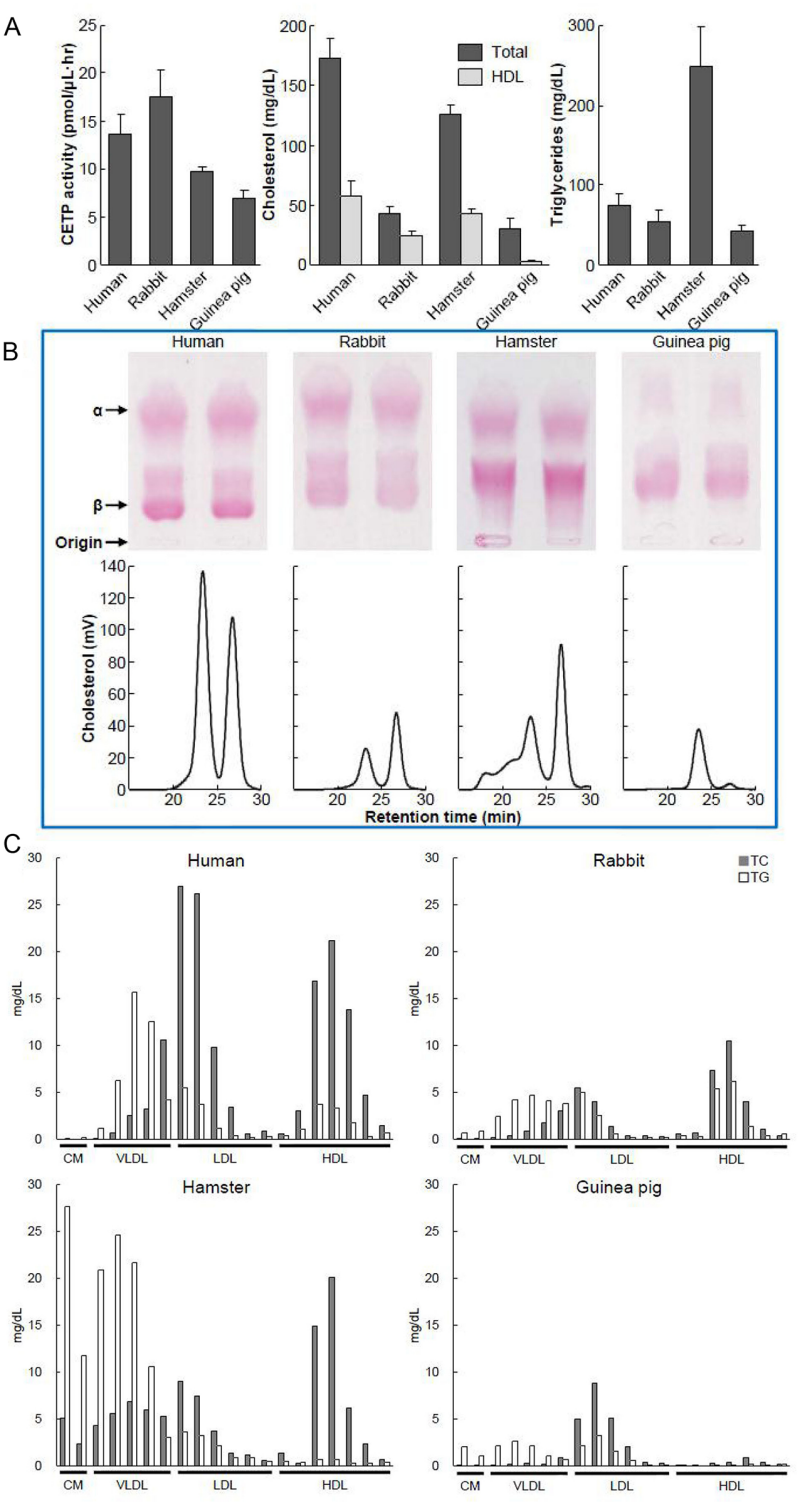
Plasma CETP activity, plasma lipids, and lipoprotein profiles. Plasma CETP activity and plasma levels of TC, HDL-C, and TG are shown in the upper panel **(A)**. Lipoprotein profiles were analyzed either by agarose gel electrophoresis (middle panel) or FPLC (bottom panel) **(B)**. Cholesterol and triglycerides in each fraction were quantitated and are shown in **C**.

We further compared the plasma CETP activity in each species along with their lipoprotein profiles. As shown in Fig 3 **and Raw Data A-B in** S2 File, the rabbit exhibited the highest plasma CETP activity among the four species: rabbit > human > hamster > guinea pig. Plasma TC levels of rabbits, hamsters, and guinea pigs were much lower than that of normal human plasma levels: hamster TC levels were about 80% of humans, but rabbits and guinea pigs were less than 25% of humans. Plasma HDL-C levels were extremely low in guinea pigs, followed by rabbits and hamsters, compared with human HDL-C. Regardless of this, the ratio of HDL-C/non-HDL-C in hamsters seemed close to that of humans. Unexpectedly, hamster plasma TG levels were 250 mg/dl on average, apparently higher than all other species. Plasma lipoprotein profiles were analyzed by agarose gel electrophoresis. Compared with human lipoproteins, rabbit lipoprotein profiles were very similar, but both α- and β-migrating lipoproteins moved faster than those of humans on agarose gel electrophoresis. In hamster lipoproteins, there was prominent accumulation of pre-β-migrating particles, whereas guinea pig α-migrating lipoproteins were almost invisible, and other particles moved to the pre-β-migrating position.

Lipoprotein profiles were further compared by HPLC and revealed that human lipoproteins were characterized by two peaks: the apoB-containing particle peak was larger than the HDL peak. Both the rabbit and hamster lipoproteins were reversed; namely, the HDL peak was bigger than the apoB-containing particle peak. Hamsters had a broad apoB-containing particle peak including both VLDL and LDL. The guinea pig HDL peak was extremely small, consistent with the agarose gel electrophoresis results shown above. Quantitation of each lipoprotein peak in all species revealed that about 70% of the cholesterol was mainly contained in apoB-containing particles in human lipoproteins, but rabbits and hamsters showed more HDL-cholesterol than non-HDL-cholesterol. Guinea pig HDL levels were low, therefore the cholesterol was mainly contained in non-HDL particles.

**Fig 4:**
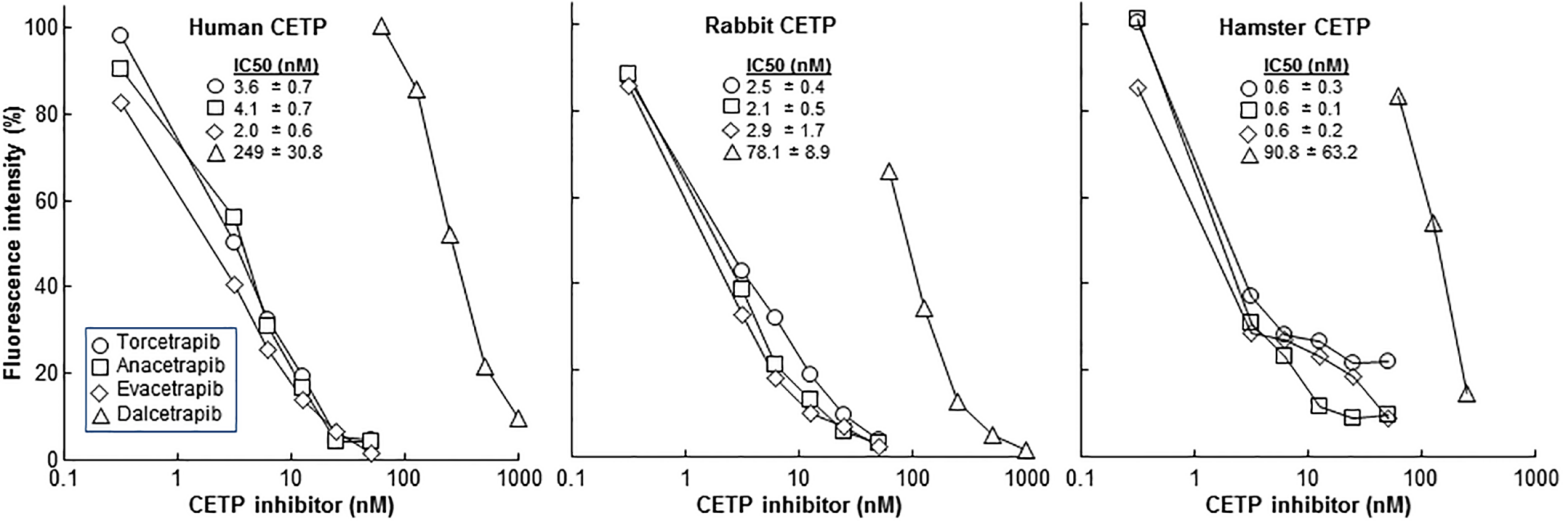
Inhibitory effects of four CETP inhibitors on plasma CETP of four species. Inhibitory effects of four kinds of CETP inhibitors on plasma CETP activity of human, rabbit, hamster, guinea pig was evaluated in vitro as described in the Materials and Methods. IC_50_ values are shown on the below.

We compared the efficacy of four CETP inhibitors (torcetrapib, dalcetrapib, anacetrapib, and evacetrapib) on the plasma CETP activity *in vitro*. For this undertaking, we performed CETP activity in the presence of each CETP inhibitor. As shown in Fig 4 **and Raw Data C in** S2 File, torcetrapib, anacetrapib and evacetrapib exhibited similar potent inhibitory activity on rabbit and hamster plasma CETP, along with human CETP, whereas dalcetrapib was much weaker than the other three inhibitors in all three CETPs. Torcetrapib, anacetrapib, and evacetrapib showed similar IC_50_ values in each CETP but dalcetrapib IC_50_ was much larger in consistence with low inhibitory effect (Fig 4). Because guinea pig plasma CETP was extremely low as shown above, it was not possible to evaluate the inhibitory effects of all CETP inhibitors. Slight inhibitory effects were seen in the presence of torcetrapib and anacetrapib but were undetectable when dalcetrapib and evacetrapib were used (data not shown).

### Binding pattern analysis

**Fig 5:**
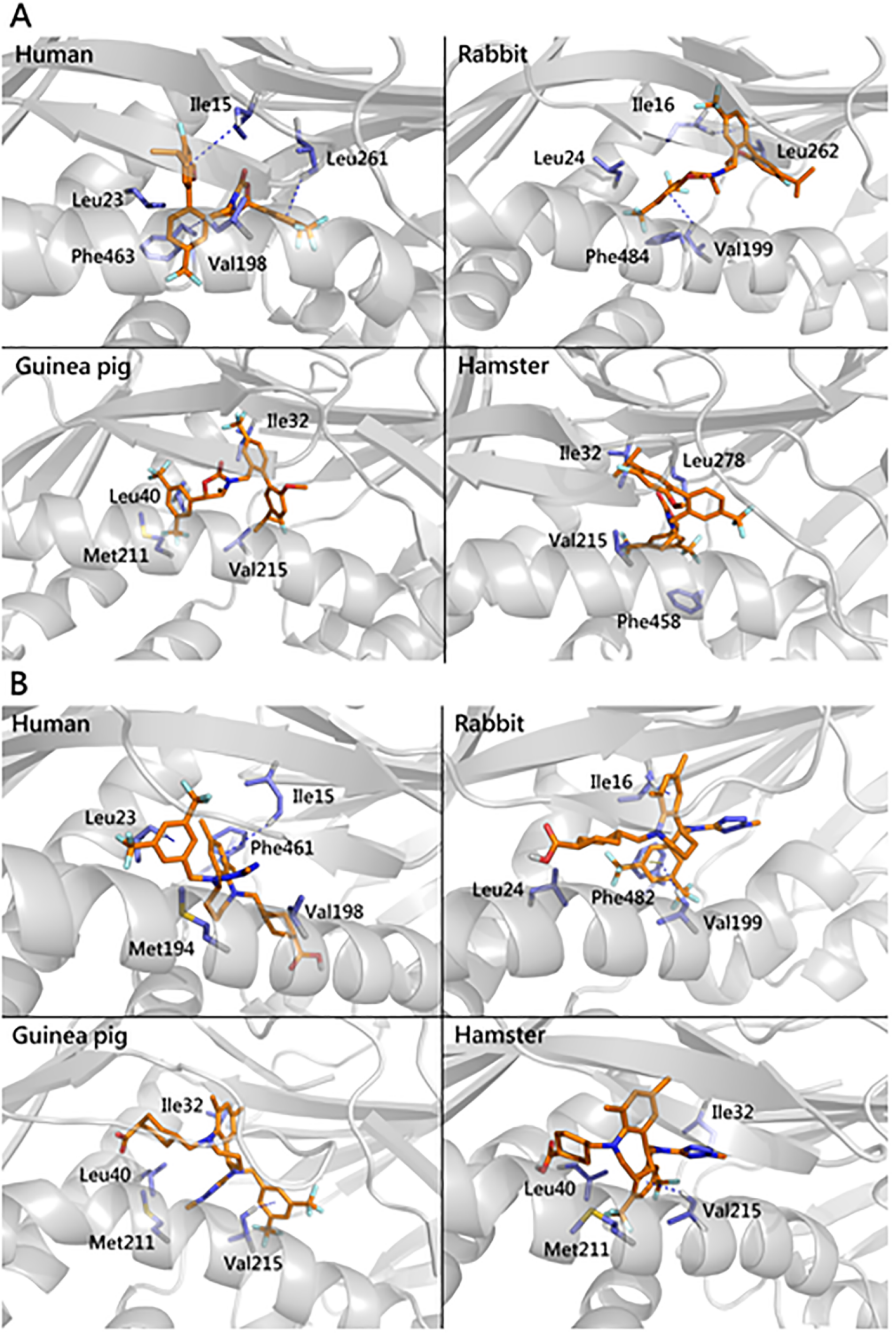
Prediction of binding patterns between two inhibitors and four proteins. Anacetrapib (A) and evacetrapib (B) are selected for evaluation of their interactions with four CETP molecules.

The optimal binding modes of evacetrapib and anacetrapib bound to each CETP molecule are shown in Fig 5. Apparently, all ligands shared the same binding pocket, which is the same as the crystal structure reported. As described in the supplemental materials **(Tables A and C in** S1 File**)**, vdW interactions were dominant in the binding modes. Anacetrapib showed a similar binding pattern when interacting with human and rabbit CETPs but showed different binding patterns when interacting with hamster and guinea pig CETPs. Multiple CH-π interactions were observed in those two complex systems. As illustrated in Fig 5A, the nonpolar residues Ile15/16 and Val198/199 in the proteins of human and rabbit CETP interacted with anacetrapib through CH-π, whereas this binding pattern was not present between anacetrapib-hamster CETP and anacetrapib-guinea pig CETP. It is well known that the CH-π interaction is weak but ubiquitous in materials and biomolecules. In these systems, there are many such interaction aggregates which may enable them to stabilize the CETP ligand binding. Similar to the anacetrapib-CETP systems, hydrophobic interactions, such as CH-π interactions and π-π interactions, play an important role in the evacetrapib-human CETP and evacetrapib-rabbit CETP complexes. The same CH-π interactions exist between the side chains of Ile15/16 and Leu23/24 and ligand aromatic rings in both human and rabbit complexes. All these similar binding patterns may support the bioassay results that the IC_50_ values are close in human and rabbit complexes.

## Discussion

In the current study, we characterized three CETP-possessing laboratory animals regarding their CETP activity, lipoprotein profiles, and CETP interactions with four known inhibitors. Although all of these animals are considered useful for the study of lipoprotein metabolism and atherosclerosis, it has not been defined whether their CETP is similar in terms of the molecular structures and interactions with the inhibitors. Biochemical analysis of plasma lipoproteins along with molecular analysis of the CETP structure and interactions with CETP inhibitors suggest that rabbits and hamsters are appropriate models for investigating CETP functions since they show similar lipoprotein profiles and CETP functions.

Laboratory rabbits originated from European rabbits (*Oryctolagus cuniculus*) and belong to the family Leporidae of the order Lagomorpha. The rabbit is an herbivore, and its typical laboratory chow diet contains ~15% protein, 40~50% carbohydrate, 2% vegetable fat, and 15~25% fiber. Normally, the cholesterol (phytosterol) content in a regular chow diet is less than 0.01%. On this type of diet, plasma cholesterol levels are in the range of 30~90 mg/dl at the age of 3~16 months[24]. Rabbits were the first and one of the best models for the study of human hypercholesterolemia and atherosclerosis because they have many lipid metabolism features (such as plasma CETP activity and intestinal-only apoB editing) that are the same as humans, and they are sensitive to a cholesterol diet and rapidly develop atherosclerosis[25]. Consistent with the previous report[19], rabbits show higher plasma CETP activity than humans, in addition to having similar lipoprotein profiles. Therefore, rabbits have been extensively used for investigating the therapeutic effects of CETP inhibitors on the inhibition of atherosclerosis[7–9, 26].

The guinea pig (Cavia porcellus) is a species of rodent belonging to the family Caviidae and the genus Cavia. Although they are still used in a lot of biological research, they are limited to a few research areas, such as juvenile diabetes, infectious disease, scurvy, and pregnancy complications, because they have been largely replaced by rats and mice in recent years. As showed in this study, guinea pigs indeed expressed detectable plasma CETP activity but at very low levels compared with humans, rabbits, and hamsters[27]. Their lipid metabolism features have been described, and some researchers even suggested the suitability of guinea pigs to study alterations to cholesterol and lipoprotein metabolism[28]. As described in the current study, the lipoproteins of guinea pigs are characterized by a high ratio of apoB-containing particles, but these particles are mainly VLDLs (pre-β-migrating) rather than LDLs. A few studies have used cholesterol-fed guinea pigs for the study of atherosclerosis in the literature, but the pathological features of the atherosclerotic lesions have not been reported in detail[29]. Therefore, it is still not clear whether this model is useful for studying atherosclerosis. In addition, there are no reports using guinea pigs as a model to evaluate CETP inhibitors. Our results shown here also indicate that guinea pigs may not be suitable for examining the efficacy of CETP inhibitors.

Gold Syrian hamsters are also rodents, like guinea pigs, but belonging to the subfamily Cricetinae, which exhibits many features similar to humans, including lipoprotein profile features, CETP expression, and intestinal-only apoB editing[30]. When they were fed with a diet containing high fructose, they developed both hyperlipidemia and insulin resistance[31]. Aortic atherosclerosis could be induced by feeding a high cholesterol diet. Therefore, hamsters are considered another model for the study of lipid metabolism and drug development[32]. However, the lesions of aortic atherosclerosis in hamsters were mild and mainly fatty streaks[33], unlike rabbits in which both fatty streaks and advanced lesions could be induced. Like guinea pigs, but unlike rabbits and mice, another drawback of the hamsters is the paucity of genetically modified models. Recently, both transgenic and knock-out hamsters have been reported[34, 35]. In the current study, we noticed that hamsters have high levels of plasma TG (<250 mg/dl) compared to other species. Although it is not clear whether high TG levels affects CETP activity or visa versa, this species seems to have higher TG levels, which was also reported by others[36, 37]. TG levels of hamsters were 162~219 mg/dl in these reports[35,36]. Therefore, it is possible that plasma levels of TG of hamsters may be quite different because they are out-bred animals.

In the current study, we also attempted to compare the binding modes of known inhibitors to CETP. As shown in Fig 5, binding modes of evacetrapib and anacetrapib bound to each CETP molecule are characterized by vdW interactions. Anacetrapib showed a similar binding pattern when interacting with human and rabbit CETPs but showed different binding patterns when interacting with hamster and guinea pig CETPs. This finding is supportive of CETP activity and the inhibitory analysis shown in Figs 3 and 4. In our human model (2OBD), Ile15 and Leu261 belong to the central β-sheet domain, Leu23 and Val198 belong to N-terminal, whereas Phe463 belongs to the α-helix X (Fig 5A). The central β-sheet domain and α-helix X are essential for tunnel mechanism and CETP functions, and the inhibitors will clog the N-terminal pocket and hinder the binding and transfer of neutral lipids[16]. Interestingly, in all models we have built, human CETP- and rabbit CETP-inhibitor complexes always have weaker interactions than hamster CETP- and guinea pig CETP-inhibitor complexes, which may help explain why IC50 values are close in human and rabbit CETPs although this conjecture remains to be verified in future.

As mentioned in the introduction, CETP has been considered as a potential target for elevating plasma HDL-C thereby treating cardiovascular disease. Our recent study using knock-out rabbits demonstrated that deletion of CETP gene in rabbits protects against cholesterol diet-induced atherosclerosis[38]. In spite of this, clinical trials so far have not shown any beneficial effects of CETP inhibitors on cardiovascular death[10–12] because inhibition of CETP indeed increases the plasma levels of HDL-C but at the same time, such inhibition may hamper the reverse cholesterol transport, an important process for HDLs to carry cholesterol from the peripheral tissues back to the liver. Clearly, further studies using appropriate animal models are required to elucidate CETP pathophysiological functions.

In conclusion, three species of laboratory animals with CETP expression were compared regarding the CETP molecular structures and functions, lipoprotein profiles, and interactions between CETP and known inhibitors. Although each species has different advantages in terms of their usefulness in lipid metabolism and atherosclerosis, rabbits as well as hamsters may be superior to guinea pigs if one aims to examine the functions of CETP and its relationship with atherosclerosis.

## Supporting information

S1 File Methods, figures and tables for *in silico* analyses. (DOCX)

S2 File Raw data. (DOC)
