# Supplementary material for: Comparative studies of three cholesteryl ester transfer proteins and their interactions with known inhibitors
Source: PLoS One. 2017 Aug 2;12(8):e0180772. doi: 10.1371/journal.pone.0180772 (PMC5540280; doi:10.1371/journal.pone.0180772)
Supplement: S1 File — (DOCX) [file pone.0180772.s001.docx]

**Supplemental Methods**

**Homology modeling**

Homology modeling was performed with 2OBD as a template, and Discovery Studio 2.5.5^1^ (DS 2.5.5) was used to generate the CETP models. The Modeller 9v4 auto-modeling strategy was then used to build ten homology models, without hydrogen atoms, for each CETP model. Accordingly, thirty models were built by optimizing the molecular probability density function, which uses a variable target function procedure in Cartesian space that employs methods of conjugate gradients and MD with simulated annealing. The model with the lowest molecular probability density function score was selected from each group, and the root mean square deviation (RMSD) value calculated for further computational study. Based on the above procedure, three initial models were constructed, and further validated by PROCHECK^2^ to confirm the model reliability **(Fig A)**.

**Homology model optimization via MD simulation**

MD simulations were employed to relax the homologous proteins of the rabbit, guinea pig and hamster. For all three CETP models, the proteins were described by AMBER 03 force field and water molecules using the TIP3P model. As for ligands, force field parameters were generated from the AMBER GAFF force field^3^, and partial atomic charges were assessed by restrained electrostatic potential charges from HF/6-31G* calculations with the Gaussian 09 package. Then, counter-ions were added to the complexes for neutralizing, and proteins were solvated in a box of TIP3P^4^ water molecules with solvent layers at 10 Å from the box edges and solute surface. Finally, topology files and the initial coordinates were generated by the *tleap* program in AMBER 12^5,6^.

A traditional procedure was adopted to run the MD simulations. First, multi-step minimization was performed. This began with minimizing all solvent molecules and restraining other atoms with 50 kcal·mol^-1^·Å^-2^. Then, restraint with 10 kcal·mol^-1^·Å^-2^ was added on protein backbone atoms. Finally, the entire complex was optimized without restraint. After minimization, the complex was heated from 0 to 300 K by Langevin dynamics for 50 ps, followed by equilibration at a constant pressure of 1 atm for 100 ps. In the heating step, 10 kcal·mol^-1^·Å^-2^ constraint was used to restrain the protein backbone atoms. The systems were relaxed about 30 ns by NVT ensemble MD simulations. During the simulations, hydrogen-containing bonds were constraint by the SHAKE^7^ algorithm and the Particle Mesh Ewald (PME) method^8^ for long-range electrostatic interactions. A cutoff of 10 Å was set for electrostatic and vdW interactions, and the time step was set as 2 fs. All MD simulations were performed with *pmemd* in AMBER 12. After 30 ns simulation, complex conformations of the last 10 ns in the stable period were clustered into a few groups. Then, representative conformations of the major cluster were chosen as the optimized model for each CETP.

In order to evaluate the performance of MD parameters, the RMSDs for backbone atoms, ligand atoms and pocket atoms (which within 8 Å from ligand) were calculated to ensure stabilization of each simulation system. As shown in **Fig B**, all RMSD values were at equilibrium after 5 ns of MD simulation. Complex conformations of the last 10 ns in the stable period were clustered into a few groups. The major conformation clusters in rabbits, guinea pigs and hamsters were 69.6%, 42.5%, 98.3%, respectively **(Fig C)**. Next, a representative conformation in the major cluster was selected as the optimized model for each CETP. Therefore, reasonable CETP structures from three species were prepared and used for subsequent assessment.

**Molecular Docking and MD simulation**

The molecular structures of anacetrapib and evacetrapib were prepared with Accelrys Discovery Studio 2.5.5^1^, including minimizing the structures in the MMFF force field^9^ and generating three dimensional conformations for each inhibitor with the CAESAR algorithm^10^. Then, the weighted Gaussian algorithm (WEGA) program^11^ was used to align anacetrapib and evacetrapib based upon cholesteryl ester extracted from the CETP complex structure (PDB ID: 2OBD); the method was 'Combo’, considering shape and pharmacophore features. The conformations with highest WEGA scores were used as the initial docking conformations.

As a benchmark of docking, the cholesteryl ester was redocked to the CETP of each species with the following: (1) AMBER12/EHT force field and London dG rescoring in MOE 2013.08^12^ (Molecular Operating Environment, Chemical Computing Group Inc.); (2) AMBER12/EHT force field and GBVI/WSA dG rescoring in MOE; (3) the extra precision (XP) mode in Glide^13-15^ of Schrödinger 2013.1; (4) the standard precision (SP) mode in Glide of Schrödinger. Evaluating the conformations with top five highest docking scores, performance of the SP mode in Schrödinger was selected as the best docking method for these systems.

The initial docking conformations of anacetrapib and evacetrapib were docked into four CETPs with the SP mode in Schrödinger 2013.1, respectively. Docking conformation results for each CETP were clustered into a few groups. The conformations with highest docking scores in the major cluster were selected and returned to the corresponding receptors as initial complexes for MD simulations.

The initial complexes were prepared in MOE 2013.08. The procedures and most parameters were similar to those of previous MD simulations. Each prepared system was simulated for 16 ns with NVT ensemble at 300K, at a constant pressure of 1 atm.

To evaluate the docking performance, four major evaluation criteria were used, including (1) ability of predicting binding poses, (2) ability of predicting affinity, (3) enrichment of virtual screening, (4) molecular diversity of hits[^1^](#_ENREF_17)^6^. In order to predict the theoretical interaction models between CETPs and their inhibitors, the ability that predicting binding poses was taken into consideration in this study. Four docking methods were employed, and the top five conformations with highest docking scores were employed to evaluate those methods. RMSDs were obtained by comparing the top five conformations and the initial one (**Fig D**). According to the results, the GLIDE-SP method was considered the most suitable method. Therefore, anacetrapib and evacetrapib were docked into the binding sites of CETPs from four species using the GLIDE-SP method. Then, a 16 ns simulation was carried out for each complex with similar procedures and parameters as described for homology structure optimization.

The RMSDs for backbone atoms, ligand atoms and pocket atoms were calculated to ensure that each simulation system was stable. RMSDs are shown in **Fig E**. All eight simulation systems became steady within 5 ns and remained stable to the end of simulation. The data indicated that the last 6 ns of each complex could be used for further analysis.

**Binding free energy calculations**

After 16 ns of MD simulation, the RMSDs of backbone atoms were employed to validate the stability of each complex. Then, 100 snapshots in the last 6 ns of the stable period were selected for binding free energy calculation using the molecular mechanics Poisson−Boltzmann surface area (MM-PBSA) method[^1^](#_ENREF_16)^7^. For each snapshot, the binding free energy Δ*G*_bind_ was calculated with equation (1), as the difference of free energy of the receptor-ligand complex (*G*_complex_) and the sum of those of the receptor (*G*_rec_) and ligand (*G*_lig_):

Δ*G*_bind_ = *G*_complex_ – ( *G*_rec_ + *G*_lig_ ) (1)

Meanwhile, Δ*G*_bind_ can be evaluated in equation (2), the difference of the sum of the molecular mechanics binding energy (Δ*G*_MM_), the solvation free energy (Δ*G*_sol_) and the solute entropic contribution at temperature T (kelvin). In equation (2), ΔG_MM_ is obtained from vdW (Δ*E*^vdw^) and electrostatic (Δ*E*^ele^) interactions of the complex (equation 3). Δ*G*_sol_ is composed of the polar contribution to solvation (Δ*G*_sol, ele_) and non-polar solvation term (Δ*G*_sol, nonpolar_) (equation 4).

Δ*G*_bind_ =Δ*G*_MM_ +Δ*G*_sol_ – *T*Δ*S* (2)

Δ*G*_MM_ =Δ*E*^vdw^ +Δ*E*^ele^ (3)

Δ*G*_sol_ =Δ*G*_sol, ele_ +Δ*G*_sol, nonpolar_ (4)

Unlike the former component, Δ*G*_sol, nonpolar_ was not assessed with the PB calculation, but determined with SASA (solvent-accessible surface area) and *γ*，*b* (experimental solvation parameters) in equation (5).

Δ*G*_sol, nonpolar_ = *γ*SASA + *b* (5)

In general, entropy calculation is required in normal mode calculations. However, it is time-consuming, and entropy differences are normally small. So, entropy contributions (-*T*Δ*S*) were ignored in order to save computational costs.

The binding affinities of anacetrapib and evacetrapib to the four CETP species were calculated to interpret the assay results of **Fig 4**. As listed in **Table A**, the binding energies of anacetrapib obtained with human, rabbit, guinea pig and hamster CETPs were -39.26, -39.64, -31.13 and -43.24 kcal mol^-1^, respectively. Meanwhile, the binding energies of evacetrapib with human, rabbit, guinea pig and hamster CETPs were, separately, -42.83, -42.31, -25.69 and -45.26 kcal mol^-1^. According to **Table A**, binding free energy was mainly attributed to vdW interactions. Obviously, binding affinities of anacetrapib and evacetrapib with guinea pig CETP were weaker than those of the other species. Moreover, the values for these two inhibitors with hamster CETP were the best, consistent with bioassay data. Considering rabbit and human CETPs, calculation results were consistent with CETP activity data that anacetrapib and evacetrapib exhibited similar inhibitory activities to rabbit and human CETPs *in vitro*.

**Clustering and analysis of CETP conformations**

The second halves of the trajectories at equilibrium were also adopted in conformation analysis. Conformations of each system at this period were clustered into a few groups, and the representative conformation from the major cluster was selected for protein conformation analysis. The complex structures were superposed with the human CETP in MOE, and the resulting RMSDs are shown in **Table B**. The RMSD values were employed to compare the representative conformations from four species with anacetrapib or evacetrapib. As shown in **Table** **B**, representative conformations of rabbit CETP had the lowest RMSD values when binding to anacetrapib or evacetrapib. On the contrary, representative conformations of the hamster CETP had the highest RMSD values. This result indicated that equilibrium conformation of the rabbit CETP is more similar to the human CETP than those of the other two species in binding with anacetrapib or evacetrapib.

**Hot residue analysis**

For better understanding the interaction between the inhibitors and CETP proteins, binding energy decompositions were employed to explore the source of important interaction energies. The calculated Δ*G*_bind_ values were decomposed into single-residue contributions by MM-PBSA method in Amber 12. As mentioned above, vdW interactions, which are similar to hydrophobic interactions, play a key role in binding affinity. Interactions shown in crystal structures are mainly hydrophobic interactions[^1^](#_ENREF_18)^8^ that contained in van der Waals contribution. Herein, the top ten key residues sorted by Δ*E*^vdw^ values for each system are listed in **Table** **C**. Comparing the human protein and those of the other three species, common residues were extracted, and their vdW energies applied for the linear fitting with the energies in human CETP complexes. In anacetrapib-CETP systems, R square values for common residues from the rabbit, guinea pig, hamster were 0.6207, 0.0236 and 0.0116, respectively. In evacetrapib-CETP systems, R square values for common residues from the rabbit, guinea pig and hamster were, separately, 0.3894, 0.0487 and 0.2354. Based on **Fig F**, rabbit systems had a high correlation to human counterparts in either anacetrapib-CETP or evacetrapib-CETP complexes, whereas other species showed no or low correlation to the human CETP. Therefore, the inhibitor and rabbit CETP complex system had the highest correlation with the human counterpart. Undoubtedly, these results suggest that the rabbit CETP is comparable to the human one, and it would be reasonable to choose rabbits for CETP assessment.

**Supplementary References**

1. Accelrys Discovery Studio, version 2.5.5; Accelrys Software Inc.: San Diego, CA, 2010.

2. Laskowski RA, MacArthur MW, Moss DS, Thornton JM (1993) PROCHECK: a program to check the stereochemical quality of protein structures. Journal of Applied Crystallography 26: 283-291.

3. Wang, J.; Wang, W.; Kollman, P. A.; Case, D. A., Automatic atom type and bond type perception in molecular mechanical calculations. J Mol Graph Model 2006, 25, (2), 247-60.

4. William L. Jorgensen, J. C., Jeffry D. Madura, Roger W. Impey and Michael L. Klein, Comparison of simple potential functions for simulating liquid water. The Journal of Chemical Physics 1983, 79, (2), 926-935.

5. Case, D. A.; Cheatham, T. E., 3rd; Darden, T.; Gohlke, H.; Luo, R.; Merz, K. M., Jr.; Onufriev, A.; Simmerling, C.; Wang, B.; Woods, R. J., The Amber biomolecular simulation programs. J Comput Chem 2005, 26, (16), 1668-88.

6. Gotz, A. W.; Williamson, M. J.; Xu, D.; Poole, D.; Le Grand, S.; Walker, R. C., Routine Microsecond Molecular Dynamics Simulations with AMBER on GPUs. 1. Generalized Born. J Chem Theory Comput 2012, 8, (5), 1542-1555.

7. Jean-Paul Ryckaert, G. C., Herman J.C Berendsen, Numerical integration of the cartesian equations of motion of a system with constraints: molecular dynamics of n-alkanes. Journal of Computational Physics 1977, 23, (3), 327–341.

8. Zhou, R. H., E; Xu, HF; Berne, BJ, Efficient multiple time step method for use with Ewald and particle mesh Ewald for large biomolecular systems J CHEM PHYS 2001, 115, (5), 2348-2358.

9. Halgren, T. A., Merck molecular force field. I. Basis, form, scope, parameterization, and performance of MMFF94. J Comput Chem 1996, 17, (5-6), 490-519.

10. Li, J.; Ehlers, T.; Sutter, J.; Varma-O'Brien, S.; Kirchmair, J., CAESAR: A new conformer generation algorithm based on recursive buildup and local rotational symmetry consideration. J Chem Inf Model 2007, 47, (5), 1923-1932.

11. Yan, X.; Li, J. B.; Liu, Z. H.; Zheng, M. H.; Ge, H.; Xu, J., Enhancing Molecular Shape Comparison by Weighted Gaussian Functions. J Chem Inf Model 2013, 53, (8), 1967-1978.

12. Molecular Operating Environment (MOE), 2013.08; Chemical Computing Group Inc., 1010 Sherbooke St. West, Suite #910, Montreal, QC, Canada, H3A 2R7, 2013.

13. Friesner, R. A.; Murphy, R. B.; Repasky, M. P.; Frye, L. L.; Greenwood, J. R.; Halgren, T. A.; Sanschagrin, P. C.; Mainz, D. T., Extra precision glide: docking and scoring incorporating a model of hydrophobic enclosure for protein-ligand complexes. J Med Chem 2006, 49, (21), 6177-96.

14. Halgren, T. A.; Murphy, R. B.; Friesner, R. A.; Beard, H. S.; Frye, L. L.; Pollard, W. T.; Banks, J. L., Glide: a new approach for rapid, accurate docking and scoring. 2. Enrichment factors in database screening. J Med Chem 2004, 47, (7), 1750-9.

15. Friesner, R. A.; Banks, J. L.; Murphy, R. B.; Halgren, T. A.; Klicic, J. J.; Mainz, D. T.; Repasky, M. P.; Knoll, E. H.; Shelley, M.; Perry, J. K.; Shaw, D. E.; Francis, P.; Shenkin, P. S., Glide: a new approach for rapid, accurate docking and scoring. 1. Method and assessment of docking accuracy. J Med Chem 2004, 47, (7), 1739-49.

16. Kellenberger, E.; Rodrigo, J.; Muller, P.; Rognan, D., Comparative evaluation of eight docking tools for docking and virtual screening accuracy. Proteins 2004, 57, (2), 225-42.

17. Hou, T.; Wang, J.; Li, Y.; Wang, W., Assessing the performance of the MM/PBSA and MM/GBSA methods. 1. The accuracy of binding free energy calculations based on molecular dynamics simulations. J Chem Inf Model 2011, 51, (1), 69-82.

18. Lloyd, D. B.; Lira, M. E.; Wood, L. S.; Kurham, L. K.; Freeman, T. B.; Preston, G. M.; Qiu, X. Y.; Sugarman, E.; Bonnette, P.; Lanzetti, A.; Milos, P. M.; Thompson, J. F., Cholesteryl ester transfer protein variants have differential stability but uniform inhibition by torcetrapib. Journal of Biological Chemistry 2005, 280, (15), 14918-14922.

**Supplemental Figures and Tables**

**
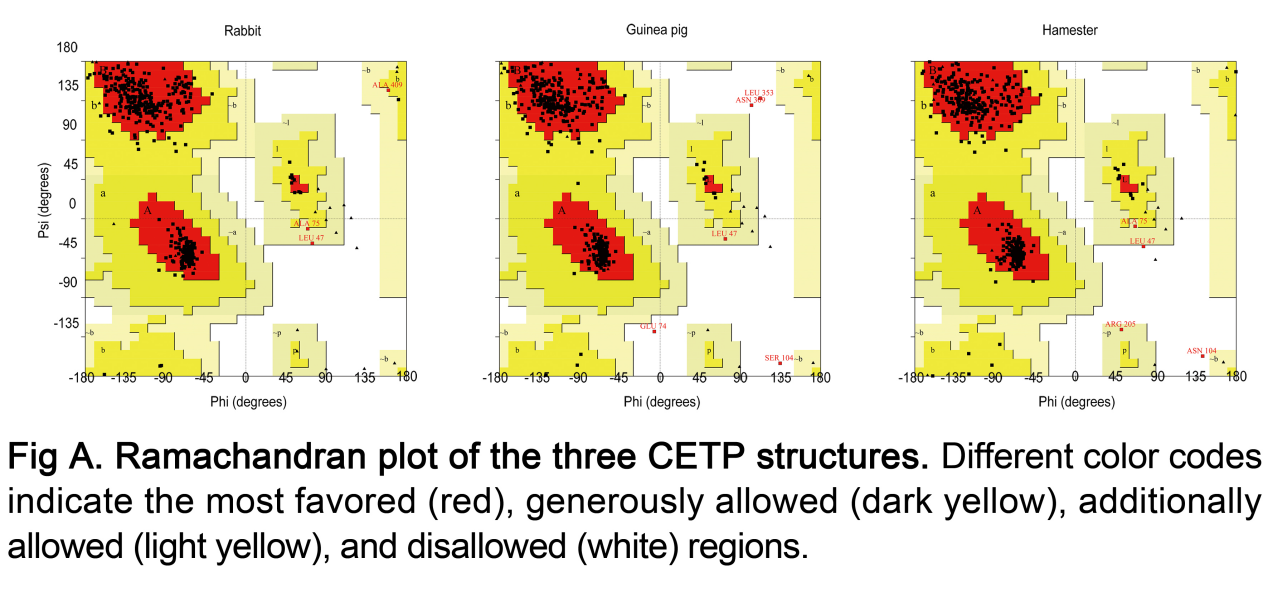
**

**
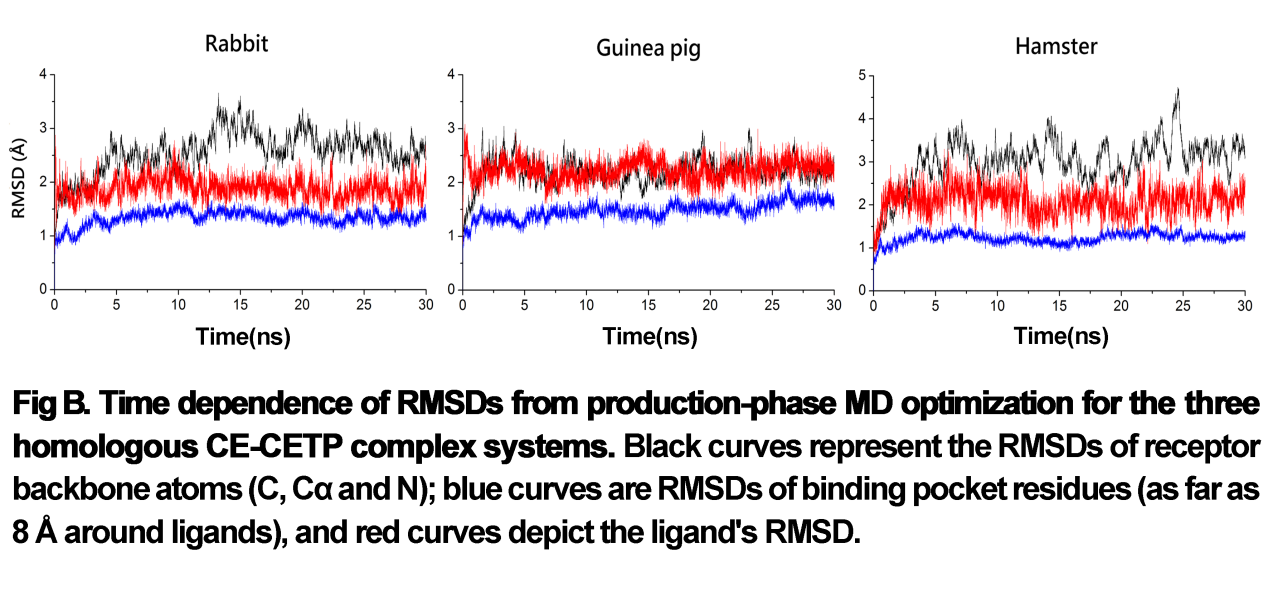
**

**
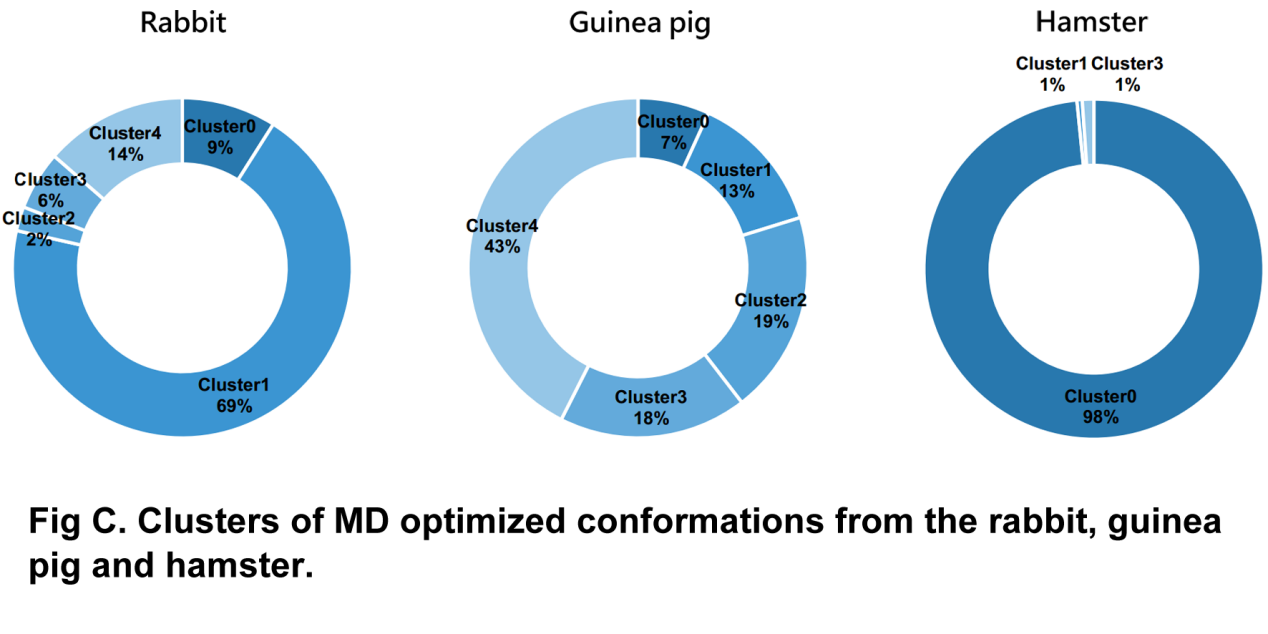
**

**
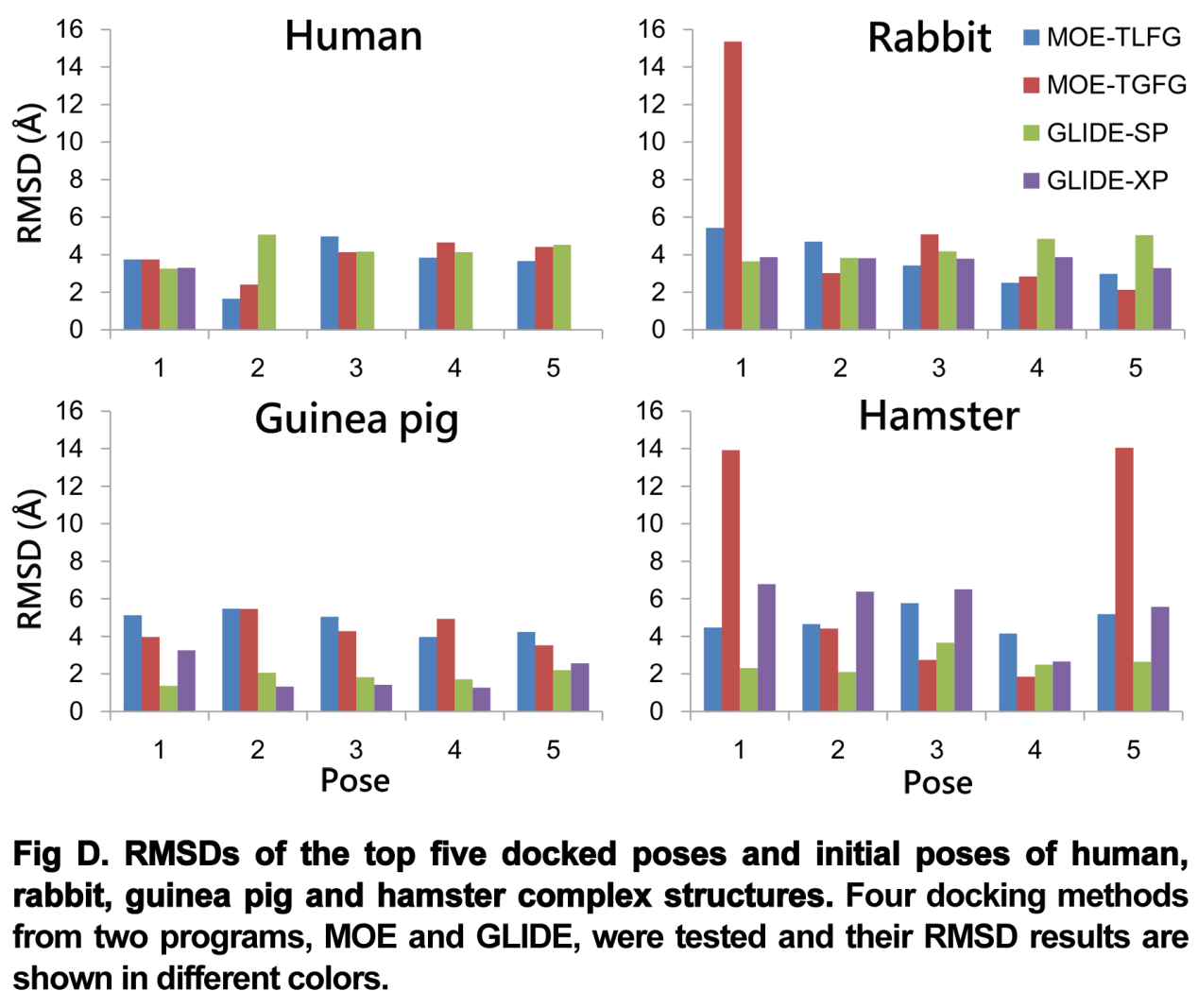
**

**
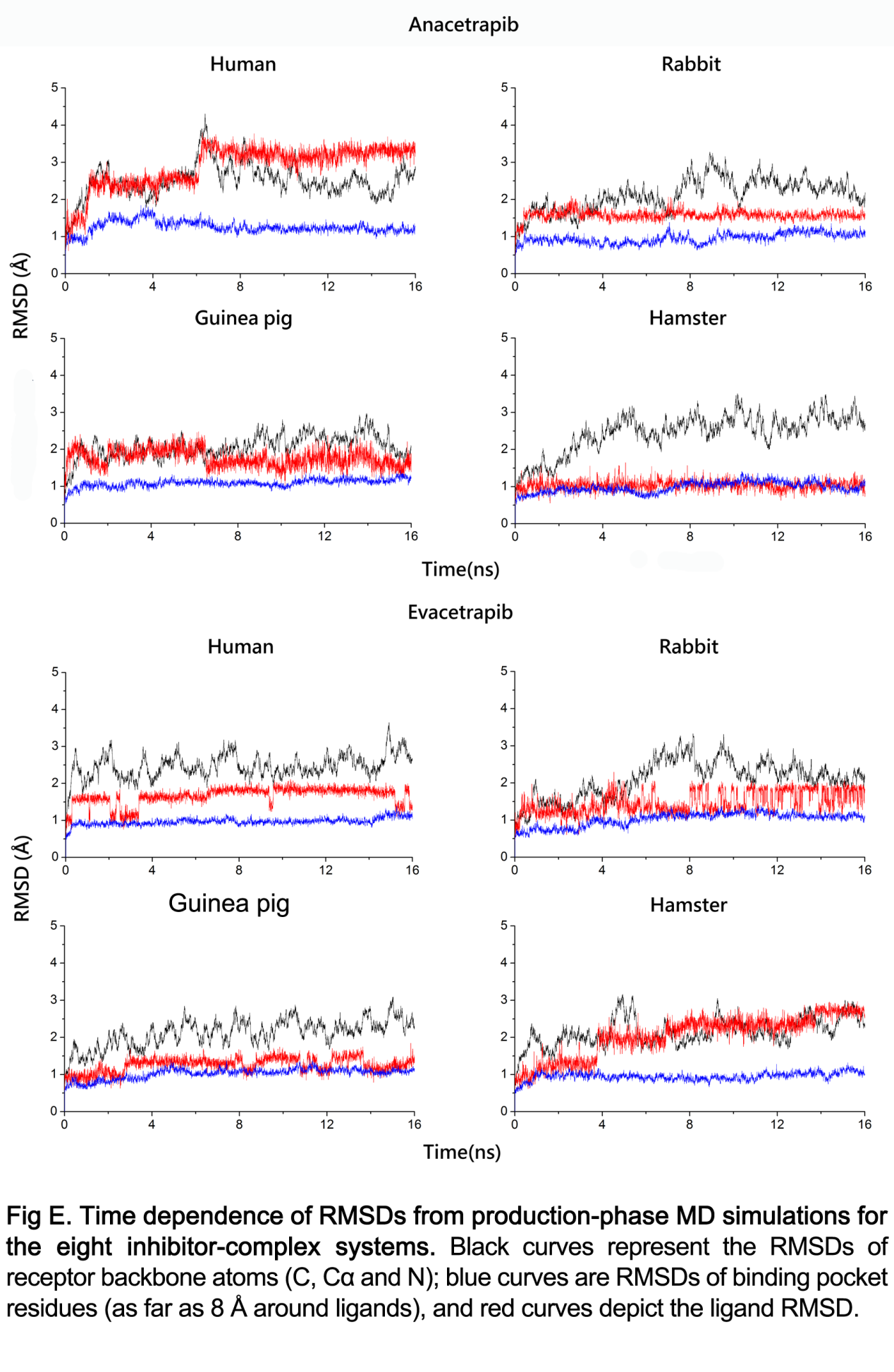
**

**
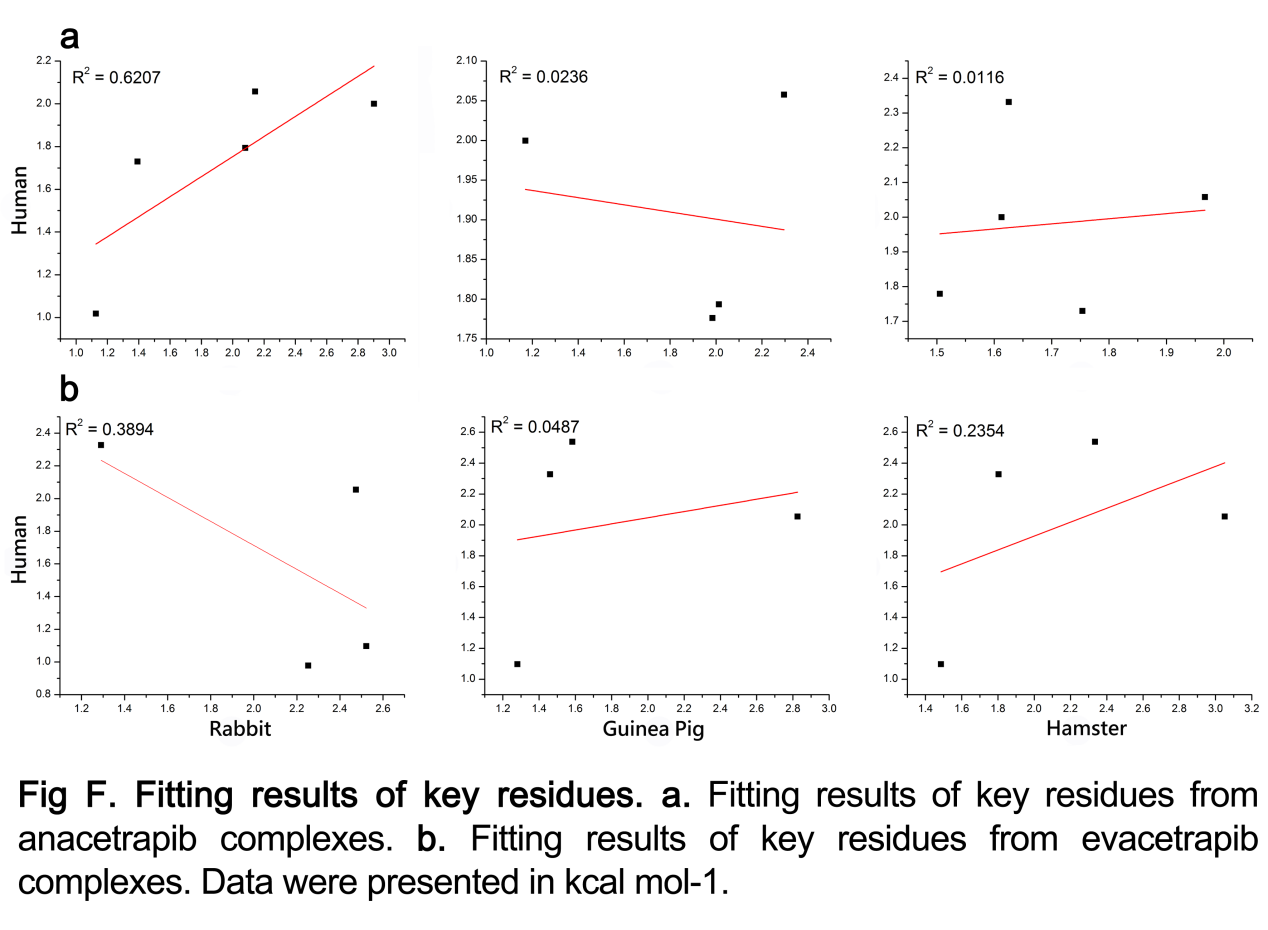
**

| **Table A. Binding energies of anacetrapib and evacetrapib bound to human, rabbit, guinea pig and hamster CETPs.** | | | | | | | | | |
| --- | --- | --- | --- | --- | --- | --- | --- | --- | --- |
| Ligand | Anacetrapib | | | |  | Evacetrapib | | | |
| Receptor | Human | Rabbit | Guinea pig | Hamster |  | Human | Rabbit | Guinea pig | Hamster |
| ΔE_VDWAALS_ | -59.80 | -64.87 | -56.09 | -66.94 |  | -63.01 | -72.49 | -69.22 | -71.78 |
| ΔE_ELE_ | -2.87 | 1.71 | -8.18 | -4.81 |  | -20.79 | -3.98 | -41.13 | -8.94 |
| ΔE_PB_ | 23.41 | 23.52 | 33.14 | 28.51 |  | 40.97 | 34.16 | 84.66 | 35.45 |
| ΔE_GAS_ | -62.67 | -63.16 | -64.27 | -71.75 |  | -83.80 | -76.47 | -110.4 | -80.72 |
| ΔE_SOLV_ | 23.41 | 23.52 | 33.14 | 28.51 |  | 40.97 | 34.16 | 84.66 | 35.45 |
| ΔE_TOTAL_ | -39.26 | -39.64 | -31.13 | -43.24 |  | -42.83 | -42.31 | -25.69 | -45.26 |

All data were presented in kcal mol^-1^.

**Table B. Representative conformations in human and other species (rabbit, guinea pig and hamster).**

| Protein-ligand | | CETP_Rabbit_ | CETP_Guinea Pig_ | CETP_Hamster_ |
| --- | --- | --- | --- | --- |
| CETP_Human_ | Anacetrapib | 2.50 | 2.79 | 3.80 |
|  | Evacetrapib | 2.96 | 3.62 | 3.24 |
| * Data were presented in angstrom | | | |  |

**Table C. Top ten energetically essential residues of rabbit, guinea pig, hamster and human CETPs and their vdW contributions to the binding energy.** **a.** Essential residues for anacetrapib. **b.** Essential residues for evacetrapib. Data were presented in kcal mol^-1^.

**a**

| **Human** | | **Rabbit** | | **Guinea pig** | | **Hamster** | |
| --- | --- | --- | --- | --- | --- | --- | --- |
| **Residue** | **Δ*E*^vdw^** | **Residue** | **Δ*E*^vdw^** | **Residue** | **Δ*E*^vdw^** | **Residue** | **Δ*E*^vdw^** |
| PHE 441 | -2.33 | ILE 16 | -2.90 | VAL 215 | -2.30 | VAL 215 | -2.64 |
| VAL 198 | -2.06 | GLN 120 | -2.57 | LEU 40 | -2.01 | ILE 32 | -2.27 |
| ILE 15 | -2.00 | VAL 199 | -2.14 | MET 211 | -1.98 | GLN 216 | -1.88 |
| LEU 23 | -1.79 | LEU 24 | -2.08 | LEU 146 | -1.65 | ALA 219 | -1.57 |
| PHE 461 | -1.78 | CYS 14 | -1.86 | PHE 479 | -1.38 | CYS 30 | -1.51 |
| MET 194 | -1.78 | ALA 196 | -1.69 | ILE 32 | -1.17 | LEU 278 | -1.25 |
| LEU 261 | -1.73 | LEU 262 | -1.39 | ALA 212 | -1.16 | LEU 245 | -1.24 |
| LEU 20 | -1.44 | PHE 484 | -1.13 | PHE 280 | -1.05 | PHE 458 | -1.23 |
| PHE 463 | -1.02 | SER 231 | -1.13 | THR 44 | -0.99 | SER 247 | -1.21 |
| LEU 468 | -0.92 | ALA 203 | -1.03 | SER 247 | -0.92 | HIE 249 | -1.05 |

**b**

| **Human** | | **Rabbit** | | **Guinea pig** | | **Hamster** | |
| --- | --- | --- | --- | --- | --- | --- | --- |
| **Residue** | **Δ*E*^vdw^** | **Residue** | **Δ*E*^vdw^** | **Residue** | **Δ*E*^vdw^** | **Residue** | **Δ*E*^vdw^** |
| MET 194 | -2.54 | **ILE 16** | -2.52 | **VAL 215** | -2.83 | **VAL 215** | -3.05 |
| LEU 23 | -2.33 | **VAL 199** | -2.47 | LEU 245 | -2.18 | LEU 146 | -2.47 |
| VAL 198 | -2.05 | **PHE 482** | -2.25 | GLN 216 | -2.08 | **MET 211** | -2.34 |
| PHE 463 | -2.03 | LEU 229 | -1.90 | **MET 211** | -1.58 | LEU 248 | -2.11 |
| PHE 441 | -1.79 | GLN 200 | -1.75 | SER 247 | -1.58 | VAL 99 | -1.81 |
| PRO 464 | -1.38 | LEU 262 | -1.74 | CYS 30 | -1.50 | **LEU 40** | -1.80 |
| THR 138 | -1.11 | CYS 14 | -1.69 | **LEU 40** | -1.46 | **ILE 32** | -1.49 |
| ILE 15 | -1.10 | ALA 203 | -1.62 | PHE 280 | -1.42 | ALA 212 | -1.33 |
| PHE 461 | -0.98 | ALA 196 | -1.41 | VAL 153 | -1.32 | LEU 37 | -1.24 |
| LEU 467 | -0.91 | **LEU 24** | -1.29 | **ILE 32** | -1.28 | SER 208 | -1.21 |
