# Supplementary material for: Comparative studies of three cholesteryl ester transfer proteins and their interactions with known inhibitors
Source: PLoS One. 2017 Aug 2;12(8):e0180772. doi: 10.1371/journal.pone.0180772 (PMC5540280; doi:10.1371/journal.pone.0180772)
Supplement: S2 File — (DOC) [file pone.0180772.s002.doc]

**Supplemental Raw Data**

Raw Data A. Plasma CETP activity and lipids for Figure 4

|  |  | CETP activity (pmol/μL/hr) | TC (mg/dL) | HDL-C (mg/dL) | TG (mg/dL) |
| --- | --- | --- | --- | --- | --- |
| Human | #1 | 12.6 | 173.2 | 59.7 | 71.1 |
|  | #2 | 11.6 | 164.5 | 56.5 | 63.2 |
|  | #3 | 13.1 | 162.6 | 73.5 | 57.8 |
|  | #4 | 16.9 | 199.5 | 61.4 | 81.6 |
|  | #5 | 14.1 | 165.2 | 39.8 | 97.8 |
|  | Mean | 13.7 | 173.0 | 58.2 | 74.3 |
|  | SD | 2.0 | 15.4 | 12.1 | 15.9 |
| Rabbit | #1 | 14.0 | 35.9 | 19.3 | 77.2 |
|  | #2 | 18.9 | 48.7 | 30.0 | 58.0 |
|  | #3 | 15.8 | 48.6 | 27.6 | 53.3 |
|  | #4 | 17.3 | 34.4 | 22.6 | 46.2 |
|  | #5 | 21.3 | 43.3 | 24.0 | 40.9 |
|  | Mean | 17.5 | 42.2 | 24.7 | 55.1 |
|  | SD | 2.8 | 6.8 | 4.2 | 14.0 |
| Hamster | #1 | 10.1 | 122.9 | 45.2 | 212.7 |
|  | #2 | 9.1 | 137.8 | 46.4 | 240.4 |
|  | #3 | 9.8 | 124.9 | 42.2 | 222.1 |
|  | #4 | 10.3 | 119.6 | 38.4 | 320.5 |
|  | Mean | 9.8 | 126.3 | 43.0 | 248.9 |
|  | SD | 0.5 | 8.0 | 3.6 | 49.1 |
| Guinea pig | #1 | 7.5 | 27.3 | 1.6 | 36.2 |
|  | #2 | 5.7 | 28.9 | 5.1 | 47.1 |
|  | #3 | 7.1 | 41.6 | 2.0 | 42.7 |
|  | #4 | 6.5 | 21.1 | 2.1 | 54.2 |
|  | #5 | 7.7 | 34.2 | 2.2 | 35.3 |
|  | Mean | 6.9 | 30.6 | 2.6 | 43.1 |
|  | SD | 0.8 | 7.7 | 1.4 | 7.9 |

Raw Data B. Density fraction analysis for Figure 5

|  |  | Human | | Rabbit | | Hamster | | Guinia pig | |
| --- | --- | --- | --- | --- | --- | --- | --- | --- | --- |
|  |  | TC  (mg/dL) | TG (mg/dL) | TC  (mg/dL) | TG (mg/dL) | TC  (mg/dL) | TG (mg/dL) | TC  (mg/dL) | TG (mg/dL) |
| CM | F1 | 0.0 | 0.1 | 0.1 | 0.7 | 5.1 | 27.7 | 0.1 | 2.0 |
|  | F2 | 0.0 | 0.2 | 0.1 | 0.9 | 2.3 | 11.8 | 0.1 | 1.0 |
| VLDL | F3 | 0.1 | 1.1 | 0.2 | 2.5 | 4.3 | 20.9 | 0.1 | 2.2 |
|  | F4 | 0.7 | 6.2 | 0.4 | 4.2 | 5.5 | 24.6 | 0.2 | 2.6 |
|  | F5 | 2.6 | 15.7 | 0.9 | 4.7 | 6.8 | 21.6 | 0.3 | 2.2 |
|  | F6 | 3.2 | 12.6 | 1.7 | 4.1 | 6.0 | 10.6 | 0.2 | 1.0 |
|  | F7 | 10.6 | 4.3 | 3.1 | 3.8 | 5.3 | 3.0 | 0.9 | 0.6 |
| LDL | F8 | 27.0 | 5.5 | 5.4 | 5.0 | 9.1 | 3.6 | 5.0 | 2.2 |
|  | F9 | 26.2 | 3.7 | 4.0 | 2.5 | 7.5 | 3.3 | 8.8 | 3.2 |
|  | F10 | 9.8 | 1.2 | 1.3 | 0.6 | 3.7 | 2.2 | 5.1 | 1.6 |
|  | F11 | 3.4 | 0.4 | 0.4 | 0.2 | 1.3 | 0.9 | 2.0 | 0.6 |
|  | F12 | 0.6 | 0.2 | 0.4 | 0.2 | 1.2 | 0.9 | 0.4 | 0.1 |
|  | F13 | 0.9 | 0.3 | 0.3 | 0.2 | 0.6 | 0.5 | 0.3 | 0.1 |
| HDL | F14 | 0.5 | 0.4 | 0.6 | 0.4 | 1.4 | 0.5 | 0.1 | 0.0 |
|  | F15 | 3.1 | 1.1 | 0.6 | 0.3 | 0.2 | 0.4 | 0.1 | 0.0 |
|  | F16 | 16.9 | 3.7 | 7.3 | 5.4 | 14.9 | 0.7 | 0.3 | 0.1 |
|  | F17 | 21.2 | 3.3 | 10.5 | 6.2 | 20.1 | 0.7 | 0.4 | 0.1 |
|  | F18 | 13.8 | 1.8 | 4.0 | 1.4 | 6.2 | 0.3 | 0.9 | 0.2 |
|  | F19 | 4.7 | 0.3 | 1.1 | 0.4 | 2.4 | 0.3 | 0.4 | 0.1 |
|  | F20 | 1.5 | 0.7 | 0.4 | 0.5 | 0.7 | 0.4 | 0.2 | 0.2 |

Rada Data C. Evaluation of CETP inhibitors on different plasma CETP activity for Figure 6

|  |  | IC_50_ (nM) | | | |
| --- | --- | --- | --- | --- | --- |
|  |  | Torcetrapib | Anacetrapib | Evacetrapib | Dalcetrapib |
| Human  CETP | #1 | 3.2 | 3.7 | 1.4 | 214.6 |
|  | #2 | 3.2 | 3.7 | 2.1 | 259.9 |
|  | #3 | 4.4 | 4.9 | 2.6 | 273.4 |
|  | Mean | 3.6 | 4.1 | 2.0 | 249.3 |
|  | SD | 0.7 | 0.7 | 0.6 | 30.8 |
| Rabbit  CETP | #1 | 3.0 | 2.7 | 2.4 | 71.7 |
|  | #2 | 2.2 | 2.1 | 1.6 | 88.2 |
|  | #3 | 2.4 | 1.7 | 4.8 | 74.4 |
|  | Mean | 2.5 | 2.2 | 2.9 | 78.1 |
|  | SD | 0.4 | 0.5 | 1.7 | 8.9 |
| Hamster  CETP | #1 | 0.9 | 0.6 | 0.4 | 163.6 |
|  | #2 | 0.4 | 0.7 | 0.8 | 56.2 |
|  | #3 | 0.5 | 0.5 | 0.6 | 52.4 |
|  | Mean | 0.6 | 0.6 | 0.6 | 90.8 |
|  | SD | 0.3 | 0.1 | 0.2 | 63.2 |
